# Assessment of Spatial Presence Experiences and Symptoms of Cybersickness in Dynamic and Static Virtual Environments

**DOI:** 10.3390/jcm14124200

**Published:** 2025-06-12

**Authors:** Witold Pawełczyk, Konrad Młyńczyk, Marcin Murawski, Anna Rutkowska, Sebastian Rutkowski

**Affiliations:** 1 Faculty of Physical Education and Physiotherapy, Opole University of Technology, 45-758 Opole, Poland; a.rutkowska@po.edu.pl; 2 Descartes’ Error Student Research Association, Faculty of Physical Education and Physiotherapy, Opole University of Technology, 45-758 Opole, Poland; s97472@student.po.edu.pl (K.M.); s94141@student.po.edu.pl (M.M.)

**Keywords:** VR, cybersickness, immersion

## Abstract

**Background/Objectives:** Virtual reality (VR) is becoming an increasingly popular therapeutic tool, with immersion being a key component that provides users with a sense of spatial presence in virtual environments. However, comprehensive research is lacking on the impact of static versus dynamic VR environments on changes in perceived spatial presence. The aim of this study was to assess the experience of spatial presence in virtual environments and to examine the relationship between spatial presence and users’ emotional responses during exposure to static and dynamic VR scenarios. The study also sought to compare the effects of static and dynamic environments on the occurrence of cybersickness symptoms. **Methods:** The study included 30 participants aged 18 to 26. Each participant was instructed to view two separate films, with each film lasting 20 min (with a 20 min break between them) in virtual reality using Oculus Meta Quest 2 VR goggles, and to share their experiences. Cybersickness was assessed using the Virtual Reality Sickness Questionnaire (VRSQ), emotional responses were evaluated using the International Positive and Negative Affect Schedule—Short Form (I-PANAS-SF), and spatial presence was measured with the Spatial Presence Experience Scale (SPES). **Results:** In the group of participants who viewed the dynamic film, a significant decrease in stress levels was observed after the projection as compared to the baseline resting state (*p* = 0.002), along with an increase in experienced relaxation (*p* < 0.001). In contrast, in the group that viewed the static film, no significant changes were found in stress levels (*p* = 0.464) or experienced relaxation (*p* = 0.455) when compared to baseline. The dynamic environment had the greatest impact on the occurrence of cybersickness symptoms, with all responses indicating a progressive increase compared to the static condition. The static environment produced only minor disturbances. **Conclusions:** These findings confirm that virtual environments can influence users’ emotional states, particularly highlighting the relationship between spatial presence and emotional experience.

## 1. Introduction

Virtual reality (VR) is a technology that enables users to interact with a three-dimensional virtual environment. In recent years, VR has developed rapidly, and with this growth, it has become an increasingly popular therapeutic tool. One of the key aspects of VR is immersion—more specifically, the sense of spatial presence, which refers to the extent to which users feel as though they are physically present within the virtual environment [1]. Immersion also refers to the sensation of being deeply engaged in a virtual environment, where the user may feel present in a different place or time [2]. Immersion in VR depends on several factors, such as the quality of graphics, sound, motion, and interactivity, and often requires a strong internet connection. For the user to fully experience the virtual environment, certain conditions must be met. A significant factor is the visual quality—the graphics must be realistic enough to evoke the sensation of being present in an entirely different place. A high level of spatial fidelity allows users to observe their surroundings in all directions, with a full 360-degree field of view [3]. Sound, on the other hand, should be properly isolated, synchronized, and spatialized with the visual content to achieve full immersion. Spatial presence is important due to its impact on user experience and the emotional responses that are triggered when transitioning into the virtual world [1]. VR enables users to enter a virtual world in which they can move around, interact with the environment, and perform various activities that may be difficult, impossible, or too dangerous to carry out in real life. Users can become fully immersed in the virtual world, with their senses—such as vision, hearing, touch, and motion—stimulated in a way that makes them feel as if they are truly present in the virtual environment. To experience all of this, VR requires specialized equipment such as headsets, gloves, motion sensors, and other devices that allow interaction with the virtual world [4].

The sense of spatial presence affects the user’s psychological state and can trigger various emotions such as joy, fear, or sadness. Therefore, another important aspect is the user themselves, who must be fully prepared to be transported to a given environment and to receive and process the stimuli they will experience [5]. A static environment in VR typically does not undergo significant changes during the user’s interaction with the virtual world. This means that there are no dynamic objects or processes that alter their appearance, state, or generate motion. An example of such a static environment could be a virtual beach featuring the sound of waves and leisure bungalows. Static environments refer to virtual settings that remain unchanged regardless of user input or interaction. Typical examples include virtual landscapes, architectural walk-throughs, or panoramic 360° environments used for relaxation or observation. Static VR environments can vary in complexity—from very simple to graphically sophisticated—but in all cases, their primary goal is to allow the user to become immersed in the virtual world and explore it without the need for interaction with dynamic elements [6].

A dynamic environment in VR includes dynamic objects or processes that change their appearance or state during the user’s interaction with the virtual world. This means that the user can interact with the environment, and in response to these interactions, the environment may change. An example of such an environment is participation in a VR-based action game, where the user controls an avatar through body movements [7]. Dynamic environments in VR typically require more advanced technologies and greater computational power to handle user interactions with the surroundings and to deliver realistic effects in real time. However, they allow the user to feel as though they are truly part of the virtual world and to explore it in a more advanced and engaging way compared to static environments [7].

However, the use of VR technology is limited by the fact that, in some individuals, it can regularly induce negative effects known as cybersickness (CS), causing symptoms such as nausea, headaches, disorientation, and visual discomfort [8,9,10]. Some individuals experience severe and prolonged nausea and discomfort even after short exposures to virtual environments [11]. Cybersickness is typically categorized as a form of visually induced motion sickness. Its exact cause is not yet fully understood. Cybersickness is typically categorized as a form of visually induced motion sickness. Its exact mechanisms are not yet fully understood. It has been hypothesized that a sensory mismatch between cues providing information about body orientation and movement plays a central role. In VR applications, optical flow may induce an illusory sense of motion, signaling to the user that they are moving in a particular direction with a certain acceleration, while their proprioceptive and vestibular systems fail to register the corresponding motion cues. This sensory conflict within the central nervous system may contribute to the onset of cybersickness. [12,13]. In general, many individuals report experiencing CS in VR, but this is not a universal rule, as some appear to be resistant to its symptoms. Much depends on individual factors such as gender, age, postural stability, existing medical conditions, and the activity of the central and autonomic nervous systems; device-related factors such as latency, flickering, and ergonomics; as well as characteristics of the virtual projection itself, including its duration [13]. Studies by Park et al. have shown that dynamic stimuli in VR increase cognitive load, which may lead to an intensification of cybersickness symptoms [14]. Additionally, Arshad et al. (2021) suggest that improving the alignment between visual and vestibular cues can reduce cybersickness symptoms, highlighting the importance of users’ emotional and sensory responses in this context [15].

In recent years, VR technology has found applications in medicine, particularly in hospital-based care. It has been shown that the use of VR headsets in hospital settings can have a positive impact on patients. One application of VR headsets in hospitals is the treatment of anxiety disorders, such as claustrophobia or fear of medical procedures. Patients who use VR during therapy can be exposed to anxiety-inducing situations in a safe and controlled virtual environment. This allows them to gradually overcome their fears and phobias. VR headsets are also used in pain therapy [16]. By providing virtual sensory stimuli such as landscapes, music, or relaxing sounds, VR headsets can help reduce patients’ pain and stress levels [17,18]. Another promising application of VR in hospital settings is in the domain of speech therapy, particularly for stroke patients. VR can be used to improve patients’ attention and reaction times, for example, by having a therapist introduce dynamic objects into the virtual scene and training the patient to respond appropriately. It is supposed that virtual reality-based cognitive interventions improve global cognitive function, executive functions, and memory in post-stroke patients. Researchers demonstrated that VR can be an effective tool in speech rehabilitation for stroke patients, enhancing their language and communication abilities [19]. Finally, VR headsets are also used for training medical personnel. This allows healthcare staff to practice various medical procedures in a safe virtual environment and gain essential experience before performing real-life interventions [20]. The use of VR headsets in hospital wards can contribute to improving patient experience and reducing their length of stay. At the same time, this technology can be utilized for the training and professional development of medical personnel [21].

Based on previous research findings, in this study, we aimed to assess whether dynamic VR environments elicit a stronger sense of spatial presence than static environments and whether a higher level of immersion is simultaneously associated with increased severity of cybersickness symptoms. Additionally, we sought to evaluate whether the potentially greater impact of dynamic environments is mediated by users’ emotional responses. These hypotheses are grounded in the observation that more engaging, dynamic stimuli can enhance both immersion and sensory load, which, in turn, affect emotional states and the severity of cybersickness [22,23]. Comprehensive studies analyzing the effects of static and dynamic environments on changes in spatial presence—while also accounting for the occurrence of cybersickness symptoms—may contribute to a better understanding and more effective therapeutic use of VR. Moreover, these insights could inform the development of future recreational and educational VR applications, where balancing immersion, user comfort, and engagement is equally important.

## 2. Materials and Methods

### 2.1. Participants

The study group consisted of 30 participants, including 20 males and 10 females. The mean age was 21.2 years (±2.23). Among the participants, 21 individuals had no prior experience with VR devices, while 9 had used such equipment before (Table 1).

### 2.2. Intervention

The device used in the study was the Oculus Meta Quest 2 VR headset (Meta Platforms, Inc., Menlo Park, CA, USA), utilizing the YouTube application. The headset allows users to view 360° video content. The study consisted of two parts: the first involved dynamic stimulation, while the second involved static stimulation.

During the 20 min dynamic condition, participants sat on a swivel chair and were “transported” into virtual roller coaster rides. These were presented as continuous segments within a single video, showing multiple roller coaster descents. The environment and its realism created the impression that the participant was actively engaged in the attraction alongside others. Throughout the projection, the participant was exposed to a dynamic, fast-changing visual field across multiple planes. Emotional intensity increased as the video progressed, along with the difficulty of the virtual challenges.

In the static condition, participants spent 20 min on a virtual beach beside a gently sounding ocean in a calm, unchanging environment. The visual scene remained uniform and static, without sudden or rapid transitions. Before the experiment began, participants rested for 20 min before the experiment, as well as after each stimulation session, participants completed a set of questionnaires. The International Positive and Negative Affect Schedule—Short Form (I-PANAS-SF) was used as a tool for the quick and effective assessment of mood and emotional state. The I-PANAS-SF consists of 10 items derived from the original 20-item PANAS questionnaire [24]. The five items assessing positive emotions are active, determined, attentive, inspired, and alert. The five items assessing negative emotions are afraid, nervous, upset, hostile, and ashamed [25,26]. To assess the level of spatial presence in participants, the SPES (Spatial Presence Experience Scale) questionnaire was used. SPES is an eight-item instrument based on a six-point Likert scale, designed to evaluate the sense of spatial presence in virtual reality. It enables the determination of the extent to which participants experienced immersion and a physical sense of being present within the simulated environment [26,27]. The Virtual Reality Sickness Questionnaire (VRSQ) was used to identify the presence of cybersickness symptoms. It consists of 16 items assessing the severity of nausea, oculomotor disturbances, and disorientation. Responses were rated using a five-point scale (0–4) [26,28].

### 2.3. Statistical Analysis

Data were collected using Microsoft Excel and subsequently analyzed using Statistica 13 (StatSoft, Kraków, Poland) and JASP (JASP Team 2022. JASP (Version 0.16.4), Amsterdam, The Netherlands). Continuous variables were presented as means ± standard deviation (SD).

Comparative analysis between the dynamic and static projections, based on the applied research tools, was conducted using the Wilcoxon signed-rank test. The level of statistical significance was set at *p* < 0.05 for all tests. Spearman’s rank-order correlation was used to assess the relationships between the SPES, PANAS, and VRSQ scales, likewise between sex and outcomes data, as it is appropriate for data that do not meet the assumptions of normality. Spearman’s rank-order correlations were interpreted using Cohen’s guidelines, where r ≈ 0.10 is considered small, r ≈ 0.30 is medium, and r ≈ 0.50 or above is large. A moderation analysis was also conducted to examine whether the type of environment (dynamic vs. static) moderates the relationship between spatial presence and both emotional responses and cybersickness symptoms. Additionally, a mediation analysis was performed to determine whether emotions (measured using the PANAS questionnaire) mediate the relationship between spatial presence (SPES) and cybersickness symptoms (VRSQ).

Sample size estimation was performed using G*Power 3.1.7 software. Calculations were conducted for a single group with eight repeated measurements, assuming a correlation of 0.5 between repeated measures. The Type I error rate was set at 5% (alpha level = 0.05), and the statistical power was set at 90% (Type II error = 10%). The non-sphericity correction was set to 1.0, and the expected effect size for the main outcomes was 0.2. Based on an anticipated 15% dropout rate, the minimum required sample size for this study was determined to be 30 participants.

## 3. Results

Analysis of responses from the I-PANAS-SF questionnaire revealed a significantly higher intensity of emotions such as hostility, alertness, nervousness, and fear during the dynamic projection compared to the static one. The total positive affect score for the dynamic projection was 12.467 (SD = 4.776), while for the static projection, it was 10.6 (SD = 4.375). The total negative affect score for the dynamic projection was 8.3 (SD = 3.395), compared to 6.2 (SD = 2.355) for the static projection. The mean total score for both positive and negative emotions was higher in the dynamic condition (Table 2).

Analysis of responses from the SPES questionnaire revealed a significant difference in the item asking whether “it seemed as if the participant could do whatever they wanted within the environment presented.” The values obtained for this item were significantly higher in the static projection condition, with a mean score of 3.367 (SD = 1.098) (Table 3).

Analysis of responses to the VRSQ revealed an increase in all assessed parameters following the dynamic projection. In contrast, the static projection resulted in increased scores only in the domains of dizziness and blurred vision. Values related to general discomfort, eye strain, and swaying with eyes closed remained similar to baseline. Notably, ratings for fatigue, difficulty concentrating, headache, and mental fog significantly decreased after the static projection compared to the initial state (Table 4).

The study analyzed correlations among three scales—SPES, PANAS, and VRSQ—in the context of dynamic and static virtual environments. The results indicate significant relationships between the sense of spatial presence, emotional responses, and symptoms of cybersickness.

In the dynamic virtual environment, a strong positive correlation was observed between the sense of physical presence in the presented environment and feelings of alertness (r = 0.54) and nervousness (r = 0.46). Similarly, the impression of being spatially transported into the virtual environment was strongly correlated with alertness (r = 0.52). The dynamic VR projection elicited significantly higher levels of negative emotions compared to the static stimulation, particularly in terms of hostility, alertness, nervousness, and fear. In the static virtual environment, a strong positive correlation was observed between the sense of physical presence in the presented environment and the feeling of being inspired (r = 0.61). Similarly, the impression of being spatially transported into the virtual setting showed a moderate positive correlation with inspiration (r = 0.59), while the sense of participating in the presentation’s action correlated with the feeling of being active (r = 0.58).

The correlation analysis between VRSQ and PANAS revealed a strong positive correlation between the total VRSQ score and negative emotions on the PANAS scale in the dynamic environment (r = 0.63), whereas this correlation was weak in the static environment (r = 0.29). Oculomotor symptoms showed a moderate positive correlation with alertness (r = 0.48), and disorientation strongly correlated with feelings of fear (r = 0.57) in the dynamic environment. The correlation between SPES and VRSQ showed a moderate negative relationship between the sense of physical presence in the presented environment and the total VRSQ score (r = −0.42), as well as between the impression of being able to move among objects and oculomotor symptoms (r = −0.38) in the dynamic environment. These correlations were weaker in the static environment. Thus, it can be concluded that the dynamic VR environment elicits stronger negative emotions and more pronounced symptoms of cybersickness, while also providing a more immersive experience. In contrast, the static VR environment is associated with more positive emotions and a lower intensity of cybersickness symptoms, which may be beneficial in therapeutic contexts where stress and anxiety reduction are the primary goals. The analyses revealed no significant association between sex and sense of presence or positive affect. Neither PANAS-PA nor PANAS-NA scores differed significantly between female and male participants (*p* > 0.10). Similarly, cybersickness symptoms (VRSQ-total) and each of its subscales showed no significant correlations with sex.

The mediation analysis revealed that negative emotions (measured using PANAS) significantly mediate the relationship between the sense of spatial presence and symptoms of cybersickness, particularly in the dynamic environment. A mediation analysis was conducted to examine whether negative emotions (measured using PANAS) mediate the relationship between spatial presence (SPES) and cybersickness symptoms (VRSQ). The analysis included estimating the indirect effect (β) and statistical significance (*p*-value). Statistical power was set at 72%. In the dynamic environment, a stronger sense of spatial presence (measured by the item “I felt as if I were physically present in the environment presented”) was associated with increased intensity of negative emotions such as alertness and nervousness (β = 0.54, *p* < 0.01). In turn, these negative emotions were positively correlated with the severity of cybersickness symptoms (β = 0.63, *p* < 0.01). The direct effect of spatial presence on cybersickness symptoms remained significant but was negative (β = −0.42, *p* < 0.05), indicating that after accounting for the mediating role of negative emotions, a stronger sense of spatial presence is associated with a lower intensity of cybersickness symptoms.

In the static environment, the mediation effect was weaker and statistically non-significant (β = 0.12, *p* > 0.05), suggesting that in this setting, emotions do not play a meaningful mediating role in the relationship between spatial presence and cybersickness symptoms.

The moderation analysis showed that the type of VR environment significantly moderates the relationship between spatial presence (SPES) and emotional responses (PANAS). In the dynamic environment, a stronger sense of spatial presence was associated with greater intensity of negative emotions (β = 0.38, *p* < 0.05), whereas in the static environment, a stronger sense of spatial presence was related to greater intensity of positive emotions (β = 0.42, *p* < 0.05). A particularly significant moderation effect was observed in the relationship between the SPES item “I felt as if I were physically present in the environment presented” and the PANAS item “alert” in the dynamic environment (β = 0.54, *p* < 0.01), as well as between the same SPES item and the PANAS item “inspired” in the static environment (β = 0.61, *p* < 0.01). The type of environment also moderates the relationship between spatial presence and symptoms of cybersickness. In the dynamic environment, a significant negative association was observed between spatial presence and oculomotor symptoms (β = −0.42, *p* < 0.05), whereas in the static environment, this relationship was weaker and not statistically significant (β = −0.23, *p* > 0.05). The analysis also showed that the type of environment moderates the relationship between emotions and symptoms of cybersickness. In the dynamic environment, there was a strong positive association between negative emotions and cybersickness symptoms (β = 0.63, *p* < 0.01), while in the static environment, this relationship was considerably weaker and not statistically significant (β = 0.29, *p* > 0.05). These findings suggest that the type of VR environment (dynamic vs. static) significantly influences the nature and strength of the relationships between spatial presence, emotions, and symptoms of cybersickness. The dynamic environment amplifies the relationship between spatial presence and negative emotions, as well as between negative emotions and cybersickness symptoms, while simultaneously weakening the direct relationship between spatial presence and cybersickness. In contrast, the static environment strengthens the link between spatial presence and positive emotions, while weakening the association between emotions and cybersickness symptoms.

## 4. Discussion

VR is considered a technology capable of eliciting specific emotional states in users [29,30]. This is due, among other factors, to the virtually limitless possibilities of creating diverse simulations and evoking a sense of presence in the projection through increasingly advanced technologies. The PANAS questionnaire may serve as an effective tool for quantitatively assessing emotional states during sensory engagement with experiences in virtual reality. It can help elucidate the relationship between specific emotional states—such as anxiety or inspiration—and the degree of perceived presence in virtual environments [31,32,33]. Based on the results obtained from the I-PANAS-SF questionnaire, it can be concluded that dynamic VR projection induces significantly greater intensification of negative emotions compared to static stimulation. A relationship was observed between dynamic stimulation and increased levels of emotions such as heightened stress and arousal, as well as between static stimulation and reduced levels of stress and anxiety, accompanied by increased calmness and relaxation. These findings support the hypothesis that virtual environments can influence users’ emotional states, particularly the relationship between the sense of spatial presence and emotional engagement.

The study results revealed significant differences in experienced relaxation and stress levels before and after viewing both dynamic and static films. After watching the dynamic film, a significant decrease in stress levels was observed (*p* = 0.002), along with an increase in experienced relaxation (*p* < 0.001). In contrast, no significant changes were found after viewing the static film, either in stress levels (*p* = 0.464) or in relaxation (*p* = 0.455). All statistically significant results were reported at the *p* < 0.05 level. Emotional states in virtual reality may be influenced by the level of presence experienced by users. However, identifying an objective tool to assess presence remains a challenge. One instrument that attempts to address this issue is the SPES [34]. When comparing participants’ sense of presence between dynamic and static stimulation, a significant difference was observed in the final item of the SPES questionnaire, which concerned the perceived freedom of action within the VR environment. No significant differences were found between dynamic and static stimulation in the remaining items of the SPES. Our findings add to the broader literature on how different types of VR content shape users’ emotional responses and sense of immersion. Notably, we observed that the static natural environment elicited significant positive emotions while simultaneously inducing a sense of immersion.

Findings from the study conducted by Sanchez-Vives and Slater (2005) indicate that the experience of spatial presence in virtual environments may influence users’ emotions by stimulating specific neural receptors in the brain [35]. The ability to induce positive emotions in controlled laboratory settings through VR can be leveraged in research on human psychology [36]. Vorderer et al. (2004) indicated that virtual environments can influence users’ levels of anxiety and stress, as well as their degree of emotional engagement in interactions with virtual objects and characters [37]. These findings suggest that investigating the relationship between the experience of spatial presence and users’ emotional states is both important and promising for the development of more advanced virtual applications designed to positively influence users’ emotional well-being. It is worth noting that these studies were conducted on diverse participant groups and employed various measurement tools, which may contribute to a more comprehensive understanding of the connection between spatial presence and emotional responses.

Emotions elicited through immersion in VR can, in turn, serve as motivating factors for achieving intended goals, facilitate relaxation, and support the regulation of negative emotions [38]. Deschling et al. reported that virtual reality technology, when integrated with naturalistic developmental behavioral interventions (NDBIs), has a positive impact on the emotional well-being, relaxation, and motivation of children with autism spectrum disorder (ASD) [39]. VR provides a safe and controlled environment that helps reduce anxiety and stress associated with social interactions. By allowing gradual exposure to emotionally challenging situations, people improve their emotional regulation skills. VR environments are inherently appealing and engaging, which enhances involvement in therapeutic activities. Gamification and interactivity further contribute to making the therapeutic process more enjoyable and tailored to each child’s individual interests [39]. It is suggested that a higher level of immersion is associated with a stronger analgesic effect [40]. A correlation has been observed between the level of immersion and the effectiveness of pain reduction in VR therapy. Additionally, it has been noted that more engaging VR tasks offer greater potential for diverting attention away from pain [41].

The present study aligns with the conclusions presented by Anderson et al., who, in their literature review, emphasized the potential of VR as a tool for diagnosing, understanding, and treating mental health disorders [42]. We observed that different types of VR environments evoke distinct emotional responses and levels of immersion, which may be of key importance in therapeutic practice. Specifically, the static natural environment elicited positive emotions and simultaneously supported a sense of presence, which may be beneficial in treating anxiety or depressive disorders. Previous studies have shown that immersive VR interventions can support emotional regulation, reduce depressive symptoms, and enhance the quality of life in individuals with mental health conditions, such as depression, schizophrenia, and bipolar disorder [42,43,44]. Our results suggest that static environments may be particularly suitable for these populations due to their calming effect and lower risk of cybersickness. In contrast, the dynamic environment—despite offering higher immersion—generated stronger negative emotions and symptoms of cybersickness, underscoring the need for careful selection of VR content depending on the therapeutic goal and patient profile. Thus, our findings reinforce the arguments made by the review authors regarding the multidimensional potential of virtual reality in the mental health domain [42].

Our findings complement the review by Freeman et al. [44], which highlighted a lack of research on the acceptability of VR and AR in social skills training for individuals with ASD. We observed that the static VR environment elicited positive emotions and a sense of immersion without increasing stress or cybersickness, suggesting higher acceptability and potential usefulness for individuals sensitive to overstimulation. These results may serve as a basis for further research on users’ subjective experiences with VR content [44].

As demonstrated in our results dynamic VR environments elicit stronger cybersickness symptoms compared to static environments, aligning with prior studies highlighting the role of sensory complexity, speed, and visual motion in inducing discomfort. Interestingly, after exposure to dynamic content, participants’ symptoms subsided following rest or static stimulation, suggesting a possible recovery effect. This supports Palmisano’s model, which emphasizes that illusory motion and visual latency contribute to sensory conflict and cybersickness [22]. Incorporating visual compensation mechanisms in VR design may help reduce these symptoms and improve user comfort.

In the present study, a negative correlation was observed between symptoms of cybersickness and the sense of presence. This finding is consistent with previous research, which suggests that increased presence may help reduce the severity of cybersickness symptoms [23,45]. It is assumed that individuals experiencing more severe symptoms are less focused on the task and more aware of imperfections in the virtual environment [46]. It is supposed that users experiencing cybersickness tend to focus more on internal sensations rather than external stimuli, which may lead to a reduced level of presence [47]. It should be noted that the literature also includes publications reporting a positive correlation between cybersickness and presence, as well as studies showing no correlation at all. However, the quantity and quality of existing research tend to support the existence of a negative correlation [48].

The study by Bonato et al. examined the impact of uni- and biaxial rotation of the visual environment on cybersickness. Nineteen participants were tasked with observing the interior of a virtual cube that continuously rotated either around the pitch axis or simultaneously around both the pitch and yaw axes. The authors concluded that the VR stimulus involving biaxial rotation induced more cybersickness symptoms compared to the uniaxial rotation condition [49]. These analyses are consistent with the present findings, which confirm that dynamic environments have a greater impact on the severity of cybersickness symptoms compared to static environments.

Despite researchers’ awareness of the challenges posed by cybersickness, both scholars and practitioners continue to explore the potential of VR and apply it in practical settings. The benefits of VR-based therapies may outweigh the complications associated with their symptoms. VR is already being used in post-stroke patients, demonstrating effectiveness in the recovery of motor functions and promoting neuroplastic reorganization in the brain [50]. Sana investigated the potential of using VR to reduce dizziness in individuals during the early phase of post-stroke recovery. By adjusting the intensity of dynamic VR games to match patients’ symptom levels—particularly their dizziness—the study achieved a significant reduction in dizziness following the therapy [51].

Future research should aim to explore how different forms of dynamic and static VR content can be optimized to balance immersion, emotional engagement, and user comfort. Specifically, further studies could investigate personalized VR adaptations, where sensory intensity and complexity are dynamically adjusted based on individual user characteristics, such as susceptibility to cybersickness or baseline anxiety levels. Additionally, examining the long-term effects of repeated exposure to both dynamic and static environments could provide valuable insights into user adaptation, desensitization, or cumulative benefits in therapeutic and educational contexts. Finally, integrating physiological measures (such as heart rate variability or galvanic skin response) alongside self-report questionnaires could enhance our understanding of the underlying mechanisms and improve the design of future VR interventions.

## 5. Conclusions

In summary, the application of virtual reality in medicine holds tremendous potential and may significantly advance support for individuals with a wide range of psychological and physical disorders. The findings of this study indicate that static VR environments are more strongly associated with positive emotions and reduced cybersickness symptoms, which suggests their suitability for therapeutic applications aimed at relaxation and emotional stabilization. This pattern may reflect a trophotropic response—characterized by physiological calming and parasympathetic activation—as opposed to the arousing, alertness-driven ergotropic response typically associated with dynamic environments.

These preliminary associations highlight the need to further investigate whether different types of virtual stimuli can reliably elicit ergotropic or trophotropic responses in users, and whether tailoring VR content to users’ behavioral tendencies (e.g., introverted vs. extraverted profiles) can enhance therapeutic outcomes. Future research should include physiological markers (e.g., heart rate variability) to validate this conceptual framework. Moreover, experimental work is needed to test whether static VR environments can be effectively used as standalone therapeutic tools, particularly in populations sensitive to sensory overload. Continued research in this field is highly valuable, including the expansion into augmented reality, and, eventually, the use of virtual stimulation in animals with behavioral disorders. This direction is justified, as ergotropic/trophotropic and extra-/introverted responses also reflect a behavioral hierarchy observed in the animal kingdom, from which many human psychological patterns originate.

This study has several limitations, primarily related to the relatively small sample size (*n* = 30) and the individual variability among participants. Although a priori power analysis using G*Power indicated that the chosen sample was sufficient to detect small to medium effects with 90% statistical power, the limited number of participants may reduce the precision of *p*-value estimation and compromise the robustness of inferential statistics, particularly in nonparametric and mediation/moderation analyses. Moreover, while data on variables such as gender, age, and prior VR experience were collected, the small sample size constrained our ability to conduct detailed subgroup analyses to explore potential moderating or confounding effects. These individual characteristics may have influenced emotional responses, the sense of spatial presence, and the severity of cybersickness symptoms. Therefore, we recommend interpreting marginally significant results with caution and encourage future studies to replicate our findings using larger and more diverse populations. This would allow for more accurate statistical modeling and improve the generalizability of results to broader clinical and non-clinical settings.

Another limitation of the present study concerns the fixed order of condition presentation: all participants were first exposed to the dynamic VR environment, followed by the static one. This non-randomized sequence may have introduced order effects, such as habituation, fatigue, or expectancy bias, potentially influencing the results. Although this decision was made intentionally to avoid the carry-over effects of reduced arousal or cybersickness symptoms in the dynamic condition, we acknowledge that counterbalancing would have strengthened the internal validity. Future studies should incorporate randomized or counterbalanced presentation orders to better isolate the effects of environmental characteristics.

## Figures and Tables

**Table 1:** Sample characteristics.

Number of participants	30
Mean age (SD)	21.2 (2.2)
Gender: male (%)	20 (67%)
Gender: female (%)	10 (33%)
First-time VR users (%)	21 (70%)
Previous VR experience (%)	9 (30%)

SD: standard deviation; VR: virtual reality.

**Table 2:** A descriptive analysis of participants’ responses to the I-PANAS-SF questionnaire, which assessed emotions and mood, was conducted, followed by a Wilcoxon signed-rank test to compare results between the static and dynamic VR conditions.

Emotion	Dynamic Projection	Static Projection	Wilcoxon Test (*p*-Value)	Effect Size
Worried	1.533 (0.937)	1.233 (0.568)	0.112	0.30
Hostile	1.233 (0.504)	1.067 (0.254)	0.037	0.408
Alert	2.433 (1.194)	1.333 (0.661)	<0.001	0.676
Ashamed	1.276 (0.521)	1.200 (0.484)	0.484	0.149
Inspired	2.433 (1.278)	2.567 (1.331)	0.637	0.09
Upset	1.900 (1.155)	1.333 (0.884)	0.021	0.428
Determined	1.800 (1.126)	1.900 (1.242)	0.780	0.058
Attentive	2.767 (1.165)	2.300 (1.291)	0.081	0.323
Afraid	2.367 (1.273)	1.367 (0.718)	0.001	0.591
Active	3.033 (1.299)	2.500 (1.306)	0.058	0.35
Positive	12.467 (4.776)	10.600 (4.375)	0.027	0.406
Negative	8.300 (3.395)	6.200 (2.355)	<0.001	0.641

**Table 3:** A descriptive analysis of participants’ responses to the SPES questionnaire, which assessed the sense of spatial presence, was conducted. The results were further evaluated using the Wilcoxon signed-rank test to compare responses between the static and dynamic VR conditions.

Spatial Presence Item	Dynamic Projection	Static Projection	Wilcoxon Test (*p*-Value)	Effect Size
I felt as if I were there in the environment shown in the presentation.	3.900 (0.923)	4.00 (1.017)	0.487	0.025
It seemed as if I was actually taking part in the action of the presentation.	3.800 (1.064)	3.567 (1.194)	0.354	0.132
I had the impression that my real location had shifted into the environment of the presentation.	3.700 (1.179)	3.733 (1.112)	0.666	0.053
I felt as if I were physically present in the environment of the presentation.	3.700 (1.179)	3.773 (1.112)	0.914	0.067
The objects in the presentation gave me the feeling that I could interact with them.	2.967 (0.999)	3.033 (1.033)	0.674	0.083
I had the impression that my real location had shifted into the environment of the presentation.	3.800 (0.961)	3.733 (1.172)	0.797	0.083
I felt as if I could move around among the objects in the presentation.	3.333 (1.093)	3.433 (1.251)	0.732	0.173
It seemed as if I could do whatever I wanted in the environment of the presentation.	2.867 (1.042)	3.367 (1.098)	0.015	0.448

**Table 4:** A descriptive statistical analysis was conducted on participants’ responses to the VRSQ, which assessed symptoms of cybersickness following exposure to static and dynamic VR environments.

Symptom	Before the Test		After Dynamic Projection		Δ Dyn-Pre	After Static Projection		Δ Dyn-Stat
	Mean	Standard Deviation	Mean	Standard Deviation		Mean	Standard Deviation	
General discomfort	1.233	0.568	2.033	1.066	+0.80	1.300	0.596	+0.73
Fatigue	2.100	0.960	2.033	1.033	−0.07	1.767	0.935	+0.27
Eye strain	1.733	0.980	2.300	0.837	+0.57	1.800	0.664	+0.50
Difficulty concentrating	1.567	0.626	1.967	0.809	+0.40	1.333	0.479	+0.63
Headache	1.267	0.691	1.500	0.861	+0.23	1.100	0.548	+0.40
Mental fogginess	1.400	0.770	1.567	0.898	+0.17	1.267	0.521	+0.30
Blurred vision	1.167	0.592	1.633	0.890	+0.4	1.300	0.535	+0.33
Swaying sensation with eyes closed	1.233	0.679	1.733	1.015	+0.50	1.233	0.430	+0.50
Dizziness	1.067	0.254	1.900	1.094	+0.83	1.233	0.430	+0.67

## Data Availability

The data presented in this study are available upon request from the corresponding author.
